# Eating disorder characteristics among Hungarian medical students: Changes between 1989 and 2011

**DOI:** 10.1556/2006.2020.00078

**Published:** 2020-11-27

**Authors:** Ferenc Túry, Pál Szabó, Szilvia Dukay-Szabó, Irena Szumska, Dávid Simon, Günther Rathner

**Affiliations:** 1 Semmelweis University, Institute of Behavioural Sciences, Budapest, Hungary; 2 University of Debrecen, Institute of Psychology, Debrecen, Hungary; 3 Eötvös Loránd University, Budapest, Hungary; 4 Medizinische Universität Innsbruck, Department Psychiatrie und Psychotherapie, Innsbruck, Austria

**Keywords:** epidemiology, eating disorders, anorexia nervosa, bulimia nervosa, time changes, medical students

## Abstract

**Background and aims:**

There are contradictory findings on time changes in the prevalence of eating disorders (EDs). The first epidemiological studies in Hungary were carried out in the late 1980s. The objective of the present study was to follow the changes in the prevalence of EDs in medical students after a period of 22 years.

**Methods:**

A questionnaire survey was conducted in 1989 and in 2010. The sample comprised medical students: 538 subjects (248 males and 290 females) in 1989 and 969 subjects (261 males and 708 females) in 2010. The questionnaire contained sociodemographic and anthropometric items, the Eating Behaviour Severity Scale, the General Health Questionnaire, the Anorexia Nervosa Inventory for Self-Rating, and the Eating Disorder Inventory (EDI). In the second wave, three subscales of the EDI-2 and the SCOFF questionnaire were added.

**Results:**

Current and desired body mass index were significantly higher in the second study. Binge eating at least once a week was reported less frequently (2.7% vs 6.8% in males, 6.1% vs 13% in females) in 2010. The proportion of subclinical anorexia nervosa was higher among females in 2011 (2.5% vs 0.3%, *P* < 0.01). Among males, the proportion of counterregulatory behaviours increased significantly (from 8.9 to 14.6%).

**Discussion and conclusions:**

The increase of the proportion of subclinical anorexia nervosa and that of male EDs may relate to the importance of the changes in the sociocultural background. Further representative studies are proposed in other countries of Central and Eastern Europe among medical students and in the general population.

## Introduction

Eating disorders (EDs) have come to prominence in psychiatry and public health over the last few decades. Cultural differences in epidemiological data caused EDs to be regarded for a long time as diseases of Western industrialised countries. This makes surveys taken outside Western countries all the more interesting. An indication of the rapid changes is the appearance of new presentations of EDs and body image disorders every five to ten years (such as muscle dysmorphia, orthorexia nervosa, or purging disorder). Cultural factors may be the reason for these changes.

Within the European Union, Central and Eastern European countries occupy a unique position deriving chiefly from historically differing political, social, and economic rather than geographic factors. For more than forty years, these societies were regulated by political dictatorships, while the culture, to a greater or lesser extent, became increasingly open to Western influences. This was also reflected in the appearance of the strongly culturally-influenced phenomena of EDs. In this study, we review the principal epidemiological data on EDs, with particular regard to the Central and Eastern European region and to changes in the characteristics of EDs over time. We focus mainly on student populations.

In a survey performed by clinical interviews on a representative sample of 18–25 year-old women in Italy, the lifetime prevalence of anorexia nervosa (AN) was found to be 2.0%, that of bulimia nervosa (BN) 4.6%, and that of binge eating disorder (BED) 0.6% (Favaro, Ferrara & Santonastaso, 2003). Hoek and Hoeken (2003) reviewed 14 two-stage prevalence studies of young women. The overall point prevalence of AN was between 0 and 0.9%, and in studies applying the DSM-III-R and DSM-IV criteria, it was 0.29% on average. Two-stage studies have found the average point prevalence of BN among young women to be around 1%.

A review of studies using various methods and types of samples (medical registers, school students, university students) in non-Western countries found that the point prevalence of AN lay in the range 0.002–0.9%; for BN, the range was 0.5–3.2% (Makino, Tsuboi & Dennerstein, 2004). It is important to note, however, that the authors challenged the methodological soundness of studies showing high prevalence.

Podar and Allik (2009) carried out a meta-analysis of 94 studies involving EDI or EDI-2. They found that non-Western participants scored higher than Western participants on most of the EDI subscales, both in normal and eating-disordered samples. The authors proposed that the high scores of non-Western cultures on EDI subscales are determined by their greater level of neuroticism.

### Central and Eastern Europe

Divided Germany offered an interesting opportunity to study sociocultural effects. Neumärker, Dudeck, Vollrath, Neumärker and Steinhausen (1992), and Steinhausen, Neumärker, Vollrath, Dudeck and Neumärker (1992) compared ED test scores in groups of patients in East and West Berlin in the 1980s. The latter group had lower scores in the Eating Attitudes Test (EAT) and various elements of the EDI. A study by Westenhoefer (2001) comparing the East and West sectors of Germany found that in the East (politically part of Eastern Europe), pathological eating attitudes were more common than in the West.

Rathner, Túry, Szabó, Geyer, Rumpold, Forgács, Söllner and Plöttner (1995) examined the prevalence of EDs among university students in Austria, the former German Democratic Republic (GDR, i.e., East Germany), and Hungary. AN was completely absent, and BN was found only among women: 1.0% in Hungary, 0.6% in Austria and none in the GDR. Subclinical AN was found only in Hungary. The frequency of subclinical BN among the female students was 3.8% in Hungary, 1.7% in the GDR and 1.9% in Austria; among the male students, it was 1.2, 0.8, and 0.4% respectively.

Krch and Drábková (1996) measured EDs in a Czech student population of 981 using questionnaires. The DSM-IV BN criteria were met by 5.7% of the women, but by none of the men. One woman (0.1%) met the AN criteria. A study in Slovenia on a large sample (*n* = 4,700) of students in higher education found that 1.1% of females and 0.1% of males suffered from BN (Tomori & Rus-Makovec, 2000). In an EAT-based survey of Croatian students (225 in grades 5–8, i.e. 10–15 age group; 525 in grades 9–12, i.e. 14–18 age group; 646 university students, i.e. 18–25 age group), 10.3% of girls scored above the threshold in the youngest group, 7.6% in 9th–12th grade group, and 11.3% in the university group (Ambrosi-Randić & Pokrajac-Bulian, 2005). In a sample of 500 randomly selected Serbian female college students, the proportion of AN was 0.2%. However, 13 students had a body mass index (BMI) of less than 17.5 (Lazarević, Batinić & Vukosavljević-Gvozden, 2016).

### Hungary

The first epidemiological studies of EDs in Hungary were carried out in 1988–1989. In a population of medical students, the proportion of the AN attitude (EAT score >29) was 1.5% among men and 3.6% among women. Interviews established the frequency of BN according to DSM-III-R as 0.8% among men and 1.3% among women (Túry, Szabó & Szendrey, 1990). In a subsequent study of secondary school students, no BN was found, but the proportion of the AN attitude among girls was 2.4% (Szabó & Túry, 1991). A repeat of the study among medical students one year later, employing more precise methods under international collaboration, found no AN, but the proportion of BN among women was 1.0%, and that of subclinical BN was 3.8% among women and 1.2% among men (see above – Rathner et al., 1995).

A questionnaire survey by Tölgyes and Nemessúri (2004) found BN prevalence of 0.6% among female secondary school and university students. Subclinical BN was measured at 4.5% among women and 0.8% among men. The first representative survey in Hungary found a point prevalence of 0.03% for AN, 0.4% for BN, 1.1% for subclinical AN and 1.5% for subclinical BN among women aged 15–24 (Szumska, Túry, Csoboth, Réthelyi, Purebl & Hajnal, 2005; Szumska, Túry, Hajnal, Csoboth, Purebl & Réthelyi, 2001). EDs occurred more frequently among students than in the general population. The greatest difference was in the prevalence of subclinical AN: students were six times as likely as non-students to meet the criteria. EDs occurred substantially more frequently in families with relatively high levels of education.

### Changes in the characteristics of eating disorders over time

In a study of Dutch primary care, the incidence of BN decreased significantly over three decades (the periods 1985–1989, 1995–1999 and 2005–2009), while the overall incidence of AN remained stable (Smink, van Hoeken, Donker, Susser, Oldehinkel & Hoek, 2016).

Keel, Heatherton, Dorer, Joiner and Zalta (2006), and Keel, Baxter, Heatherton and Joiner (2007) performed a 20-year longitudinal study in the USA, on body weight, dieting and ED symptoms in 1982, 1992 and 2002. They found significant increases in body weight among both men and women. Attention to body weight and frequency of dieting decreased among women but increased among men. Women showed a far more pronounced decrease in the frequency of ED symptoms with age than did men, and these symptoms showed parallels with attention to body weight and dieting. The prevalence of BN decreased significantly during the study period. The prevalence at the three time points was, for women, 4.2, 1.3, and 1.7% respectively, and for men, 1.1, 0.4, and 0% respectively. In their interpretation, the authors proposed that these decreases may be due to decreasing attention to body weight and frequency of dieting.

An interesting survey was carried out among medical students in Germany (Weigel, Hofmeister, Pröbster, Brähler & Gumz, 2016). There were 316 medical students (232 female and 84 male) from the newly formed German states who were assessed by the EDI-2 and the General Health Questionnaire-28. Their data were compared to a historical sample of East German medical students before the German reunification. Significantly higher levels of drive for thinness and body dissatisfaction were found among female students 20 years after German reunification. No significant changes in eating pathology were observable among male medical students. The authors suggest that acculturation to Western beauty ideals is more likely to affect female medical students.

## Objectives

The objective of our study was to assess the epidemiological changes of EDs in Hungary over a period of two decades. This also has a transcultural significance, considering the special sociocultural background of Central and Eastern Europe.

Recent decades have brought many sociocultural changes. The fundamental political changes in the region led to enhanced adoption of Western European values compared with the period of socialist regimes, when Western ideals spread informally.

Our hypothesis was that the general morbidity of EDs will increase over time. An increase in the morbidity of male EDs, the frequency of obesity, and in subclinical EDs can be expected.

## Methods

### Participants

The study populations and the methods used to assess the proportion of ED syndromes in the second study wave were made as similar as possible to those of the first.

In 1989, medical students took part in an epidemiological study in Debrecen (Hungary), Leipzig (German Democratic Republic) and Innsbruck (Austria). The study was repeated in Hungary in the periods May–August 2010 and May–October 2011. As in the first study wave, the study samples in 2010 and 2011 comprised medical students. However, in addition to students of Debrecen University (Hungary), the repeated study was extended to medical students of Semmelweis University in Budapest (Hungary).

In 1989, a total of 538 medical students – 290 women and 248 men – took part in the study in Debrecen University. The data was gathered using a paper-based self-completed questionnaire. In 2010 and 2011, the questionnaire was completed by 969 medical students, 708 women and 261 men. In Semmelweis University in Budapest, an online questionnaire was used (*n* = 462), and in Debrecen University, the questionnaires were completed either on paper (*n* = 192) or online (*n* = 315). There was no duplication of data. The basic demographic data of the samples are presented in Table 1.

**Table 1.** Demographic characteristics of the Hungarian sample

	Females			Males		
	1989 (*N* = 290) Mean (s.d.)	2011 (*N* = 708) Mean (S.D.)	*t* (df)	1989 (*N* = 248) Mean (s.d.)	2011 (*N* = 261 Mean (S.D.)	*t* (df)
Age (yr)	21.9 (3.6)	22.4 (2.3)	2.19* (389.3)	20.9 (2.1)	22.6 (2.4)	7.35*** (309.1)
Height (cm)	167.1 (5.7)	168.0 (6.3)	2.20* (590.2)	179.5 (6.5)	181.1 (6.7)	2.39** (334.4)
Weight (kg)	57.1 (7.2)	60.9 (11.5)	6.29*** (835.6)	72.6 (9.1)	76.8 (13.5)	3.47*** (254.0)
Current BMI	20.5 (2.3)	21.6 (3.9)	5.52*** (874.7)	22.5 (2.2)	23.4 (3.6)	2.85** (237.9)
Highest BMI (lifetime)	21.9 (2.7)	23.1 (4.6)	5.12*** (877.6)	23.3 (2.5)	24.8 (4.4)	3.93*** (227.6)
Lowest BMI (as adult)	19.0 (2.0)	19.9 (3.0)	5.53*** (792.1)	21.2 (2.3)	21.7 (2.9)	1.84* (286.4)
Desired BMI	19.6 (1.2)	20.1 (2.0)	4.85*** (863.7)	22.7 (1.7)	23.2 (2.2)	2.45** (280.8)

Significant differences between 1989 and 2011: * *P* < 0.05; ** *P* < 0.01; *** *P* < 0.001.

BMI: body mass index.

### Measures

In 1989, the instruments included various self-report questionnaires: sociodemographic and demographic data. There were also questions concerning current and desired weight, height, and menstrual status. The questionnaire incorporated the 28-item version of the General Health Questionnaire (GHQ – Goldberg & Hillier, 1979), the Anorexia Nervosa Inventory for Self-Rating (ANIS – Fichter & Keeser, 1980), the Eating Disorder Inventory (EDI – Garner, Olmstead & Polivy, 1983) and the Eating Behaviour Severity Scale (EBSS – Yager, Landsverk & Edelstein, 1987). The Hungarian adaptation of the questionnaires was used (Túry, Sáfrán, Wildmann & László, 1997).

The GHQ is a widely used screening tool for non-psychotic psychiatric problems and has been applied worldwide with good sensitivity and specificity (Goldberg & Williams, 1988). The GHQ data are not analysed in this study.

The ANIS is a German-language questionnaire designed for diagnostic purposes and longitudinal assessment of therapy in AN (Fichter & Keeser, 1980). It contains six factors to broaden the focus from mere eating-related symptoms to central psychopathological features of AN. It is not suitable for the detection of BN. It has been administered in epidemiological studies using a cut-off point of ≥65 for high-risk case detection and a subthreshold score of 46–64 for cases at risk (Fichter, Elton, Sourdi, Weyerer, & Koptagel-Ilal, 1988; Rathner & Messner, 1993; Rathner et al., 1995; Hungarian version:; Túry, Kollár & Szabó, 1991).

The EDI is one of the most frequently used self-rating instruments for assessment of disturbed eating attitudes and behaviour and the central psychopathology found in patients with EDs. It contains eight subscales and has been used in clinical trials and epidemiological screening surveys. To detect cases at high risk a cut-off point for the drive for thinness subscale of ≥14 was suggested (Garner et al., 1983). For cases at risk, a subthreshold score of 10–13 has been used (Rathner et al., 1995).

The modified version of the EBSS used in the study assesses the frequency of disturbed eating and purging behaviour for the previous four weeks and the previous six months. It had previously been used in an Austrian study of self-help groups for women with BN (Rathner & Messner, 1993).

For the repeat survey, the authors carried out some minor modifications to the questionnaire battery used in the first wave. Three new subscales were added: Asceticism, Impulse Regulation and Social Insecurity of EDI-2 (Garner, 1991), the SCOFF questionnaire, a 5-item screening tool for the assessment of EDs (Morgan, Reid & Lacey, 1999), and the CAGE questionnaire (Mayfield, McLeod & Hall, 1974) for the detection of alcohol addiction. However, the SCOFF and CAGE data are not analysed in this study.

Simulated DSM-III-R diagnoses of EDs were generated according to the procedure in 1989. A diagnosis of AN was simulated by BMI ≤ 17.5 and an absence of at least three consecutive menstrual cycles (for women). To approach the characteristic psychopathology of AN/BN the suggested cut-off scores for the ANIS total score (ANIS ≥ 65) or that of the EDI drive for thinness subscale (EDI-DT ≥ 14) were used. For the diagnosis of AN, all the above criteria had to be present. For a diagnosis of BN, a minimum of two binge episodes a week for at least four weeks (EBSS), an above cut-off EDI drive for thinness subscale or ANIS total score, and at least one compensatory behaviour (vomiting, use of laxatives/diuretics/diet pills, strict dieting according to the EBSS) had to be present on a weekly basis, throughout the last four weeks. For subclinical syndromes, ED not otherwise specified (DSM-III-R) was used, as in the study of Rathner and Messner (1993). Criteria for subclinical AN were a BMI below 19, either irregular menstruation or amenorrhoea, and at least one sub-threshold test score (ANIS: 46–64, EDI-DT: 10–13). Subclinical BN was diagnosed if binge eating episodes occurred at least once a week over the past four weeks, a sub-threshold test score (see above) was reported, and compensatory behaviour was present at least fortnightly over the last four weeks (Rathner et al., 1995). Clinical and subclinical AN according to DSM-5 was simulated similarly as mentioned above, leaving out amenorrhoea as a criteria. Clinical BN according to DSM-5 was defined with the following criteria: minimum one binge eating episode per week for at least three months, at least one compensatory behaviour for a minimum of three months, ANIS ≥ 65 or EDI-DT ≥ 14 and EDI bulimia subscale ≥ 14. Subclinical BN according to DSM-5 was defined with the following criteria: binge eating episodes, less than once a week, for three months; compensatory behaviour, less than once a week, for three months; ANIS total score: 46–64, or EDI-DT score: 10–13; and EDI bulimia score 6–13.

### Procedure

Participation was voluntary and anonymous. However, many students gave their names and addresses in 1989. In the first study wave, printed questionnaires were distributed among the medical students. In the second study wave in 2010 and 2011, both printed and online questionnaires were used in Debrecen University. In Budapest, only the online version of the questionnaire was used. The printed and the online versions were identical.

The diagnostic system in use at the time of the first wave of the study was DSM-III-R, and so this was also used in the diagnosis of EDs by questionnaire in the second wave. The analysis, however, also used the diagnostic criteria of the system in use today, DSM-5 (American Psychiatric Association, 2013).

### Statistical analysis

We carried out descriptive statistics on the data, calculated averages and proportions, as in the study twenty years previously. For the proportions, we gave Agresti–Coull interval estimates as recommended by Brown, Cai and DasGupta (2001). The data, taken at different times, were compared using Welch's test and – for the proportions – the *Z*-test.

### Ethics

Ethical approval of the study: TUKEB 82/2009, Semmelweis University, Budapest. The participants gave their informed consent.

## Results

In both the male and female samples, there were significant rises in age and anthropometric parameters between the first and second surveys. The average body weight of women in the samples increased from 57.1 to 60.9 kg, i.e., by 3.8 kg; the increase for men was from 72.6 to 76.8 kg, i.e., by 4.2 kg. Both changes were significant (*P* < 0.001). Together with body weight, BMI and the highest and lowest BMI, showed significant increases for both sexes. The desired body weight also increased. Nonetheless, the BMI calculated from desired body weight for women was 20.1, still in the lower third of the normal (18.5–25.0) range, while for men, it shifted to the upper third (23.2).

The frequency of risk cases is shown in Table 2.

**Table 2.** Frequency of (high-)risk cases (frequencies in %)

	Females			Males		
	1989 (*N* = 290)	2011 (*N* = 708)	*Z*	1989 (*N* = 248)	2011 (*N* = 261)	*Z*
ANIS ≥65	13.4	17.4	1.56	1.2	4.3	2.12*
EDI-DT≥ 14	8.2	9.2	0.50	0.0	1.1	1.66
Binges> 1/wk	7.2	3.7	−2.37*	3.2	1.5	−1.27
Compens. behav.	22.1	26.3	1.39	6.0	10.0	1.66
Amen. ≥ 3 m	1.0	2.7	1.66	–	–	–
BMI ≤17.5	4.8	4.4	−0.28	0.8	1.2	0.45
ANIS 46–64	23.4	28.1	1.52	11.7	22.3	3.17**
EDI-DT 10–13	6.2	6.2	0.00	0.8	1.1	0.35
Binges 1/wk	5.8	2.4	−2.70**	3.6	1.2	−1.78
Compens. behav.	32.8	35.3	0.75	8.9	14.6	1.99*
Irreg. menstr.	34.7	23.8	−3.52***	–	–	–
BMI 17.6–18.9	20.7	14.5	−2.41**	3.6	5.8	1.17

Note: ANIS: ANIS total score; EDI-DT: EDI Drive for Thinness score; Binges: EBSS binge eating score; Compens. behav.: compensatory behaviour (EBSS); Amen.: amenorrhoea; Irreg. menstr.: irregular menstruation.

Significant differences between 1989 and 2011: * *P* < 0.05; ** *P* < 0.01; *** *P* < 0.001.

High-risk cases: at least one compensatory behaviour to avoid weight gain (vomiting, laxatives, diuretics, slimming pills, dietary restrictions), at least once a week, and persisting at least four weeks.

Risk cases: at least one compensatory behaviour, at least once in two weeks, and persisting at least four weeks.

The proportion of individual EDs changed over the twenty years. The occurrence of bingeing decreased. In the case of women, this was a significant change: high-risk, several binges per week were reported by 7.2% of female respondents in 1989 and 3.7% in 2011 (*P* < 0.05), and the occurrence of bingeing once a week changed from 5.8 to 2.4% (*P* < 0.01). The frequency of binges recorded among the men also decreased: the percentage bingeing several times per week fell from 3.2 to 1.5%, and the percentage bingeing once per week fell by two thirds, from 3.6 to 1.2%, but these differences were not statistically significant.

The occurrence of irregular menstruation showed a marked decrease, from 34.7 to 23.8% (*P* < 0.001). Another pronounced change among women was that the proportion in the BMI range 17.6–18.9 decreased from 20.7 to 14.5% (*P* < 0.01).

The proportions of men with ANIS scores in both the risk and high-risk ranges increased significantly: 1.2% of men scored over 65 points in the ANIS test in 1989 as against 4.3% in 2011 (*P* < 0.05); the change for scores between 46 and 64 points was from 11.7 to 22.3% (*P* < 0.01). In addition, the proportion of men who reported compensating behaviours once per week also increased – from 8.9% in 1989 to 14.6% in 2011 (*P* < 0.05).

The simulated clinical and subclinical proportion figures are given in Table 3. These were calculated in two ways: firstly, according to DSM-III-R, enabling a comparison with the 1989 figures, and secondly, according to the current DSM-5 system. This showed the differences between the two methods of calculation.

**Table 3.** Simulated DSM-III-R diagnoses of clinical and subclinical eating disorders (estimated point prevalence in 1989 and in 2011 in %, 95% CI in parentheses)

	Females					
	*DSM-III-R* 1989 (*N* = 290)	*DSM-III-R* 2011 (*N* = 708)	*Z*	*DSM-III-R* 1989 (*N* = 290)	*DSM-5* 2011 (*N* = 708)	*Z*
AN	0.0 (0.00–1.27)	0.0 (0.00–0.67)	0.00	0.0 (0.00–1.27)	0.3 (0.02–1.14)	0.93
BN	1.0 (0.20–2.95)	1.6 (0.88–2.87)	0.73	1.0 (0.20–2.95)	2.4 (1.48–3.85)	1.44
Subclinical AN	0.3 (0.20–2.95)	2.5 (1.56–3.97)	2.34**	0.3 (0.20–2.95)	6.8 (5.16–8.92)	4.31***
Subclinical BN	3.8 (1.95–6.72)	2.1 (1.25–3.49)	−1.53	3.8 (1.95–6.72)	4.0 (2.78–5.74)	0.15
Clinical and subclinical ED	5.1 (2.86–8.33)	6.2 (4.38–7.91)	0.67	5.1 (2.86–8.33)	13.5 (10.08–14.94)	3.84***


	Males					
	*DSM-III-R* 1989 (*N* = 248)	*DSM-III-R* 2011 (*N* = 261)	*Z*	*DSM-III-R* 1989 (*N* = 248)	*DSM-5* 2011 (*N* = 261)	*Z*
AN	0.0 (0.00–1.27)	0.4 (0.00–2.43)	1.00	0.0 (0.00–1.27)	0.4 (0.00–2.43)	1.00
BN	0.0 (0.00–1.47)	0.0 (0.00–1.80)	0.00	0.0 (0.00–1.47)	1.1 (0.22–3.46)	1.66
Subclinical AN	0.4 (0.01–2.22)	1.5 (0.45–4.01)	1.27	0.4 (0.01–2.22)	1.5 (0.45–4.01)	1.27
Subclinical BN	1.2 (0.25–3.49)	0.4 (0.00–2.43)	−1.02	1.2 (0.25–3.49)	1.9 (0.70–4.55)	0.64
Clinical and subclinical ED	1.6 (0.44–4.07)	2.3 (0.96–5.08)	0.57	1.6 (0.44–4.07)	4.9 (2.89–8.47)	2.09*

Significant differences between 1989 and 2011 (base value for comparison DSM-III-R 1989): * *P* < 0.05; ** *P* < 0.01; *** *P* < 0.001.

AN by the DSM-III-R criteria was not found in either the 1989 or the 2011 samples. Clinical BN in the female sample occurred at the rate of 1.0% in 1989 and 1.6% in 2011. The difference was not significant. Among men, we found no BN of clinical severity in either survey. Using DSM-III-R criteria, the proportion of subclinical AN cases among women rose from 0.3% in 1989 to 2.5% in 2011 (*P* < 0.01). There was also an increase in the level of subclinical AN among men (from 0.4 to 1.5%), but this was not significant.

Among both men and women, the occurrence of subclinical BN cases decreased. For women, we found it to be 3.8% in 1989 and 2.1% in 2011, and for men, the figures were 1.2 and 0.4% respectively. Statistically, however, the difference cannot be regarded as significant.

The increase in the proportion of subclinical AN calculated using the DSM-5 criteria was also significant and was greater in size than the increase calculated using the DSM-III-R criteria: the figure for women in 2011 was 2.5% according to the old system and 6.8% (*P* < 0.001) according to the new. In the other categories (clinical AN and BN, and subclinical BN), we did not find significant differences in the proportions calculated using the two systems of classification.

Table 4 shows the EDI scores.

**Table 4.** Comparison of Eating Disorder Inventory scores

	Females			Males		
	1989 (*N* = 290) Mean (s.d.)	2011 (*N* = 708) Mean (s.d.)	*t* (df)	1989 (*N* = 248) Mean (s.d.)	2011 (*N* = 261) Mean (s.d.)	*t* (df)
Drive for thinness	3.8 (5.2)	3.8 (5.3)	0.00 (546.8)	0.8 (1.9)	1.3 (2.7)	2.43* (467.9)
Bulimia	1.1 (2.0)	1.2 (2.8)	0.63 (743.4)	0.6 (1.2)	0.6 (1.3)	0.00 (506.6)
Body dissatisfaction	8.7 (7.0)	7.9 (7.7)	−1.59 (587.5)	4.5 (4.7)	3.9 (5.7)	−1.30 (497.2)
Ineffectiveness	4.2 (5.1)	4.1 (4.7)	−0.29 (500.3)	2.2 (3.2)	3.5 (4.3)	3.88*** (479.8)
Perfectionism	5.7 (3.8)	7.4 (4.6)	6.02*** (645.1)	6.1 (3.9)	6.7 (4.0)	1.71 (506.7)
Interpersonal distrust	2.6 (3.1)	2.3 (3.3)	−1.36 (569.4)	3.0 (3.0)	2.6 (3.5)	−1.39 (501.7)
Interoceptive awareness	3.4 (3.6)	3.0 (4.2)	−1.52 (622.0)	1.9 (2.4)	2.1 (3.3)	0.78 (475.1)
Maturity fears	5.6 (4.7)	5.0 (5.0)	−1.80 (569.1)	4.6 (4.1)	4.6 (4.7)	0.00 (503.4)
Total score	35.1 (20.1)	34.7 (25.4)	−0.26 (673.0)	23.7 (13.1)	25.2 (18.7)	1.05 (466.8)

Significant differences between 1989 and 2011: * *P* < 0.05; ** *P* < 0.01; *** *P* < 0.001.

There was little change in the overall EDI score over twenty years. For women, the perfectionism subscale score showed a significant increase (from 5.7 to 7.4 [*P* < 0.001]), and for men, the score for items concerning feelings of inadequacy and personal effectiveness increased (from 2.2 to 3.5 [*P* < 0.001]).

## Discussion

The presentation of EDs and body image disorders are constantly changing. In addition to shifts in prevalence, new variations are frequently reported. It is the task of epidemiology to prepare the healthcare system for anticipated demands on its capacity by determining the prevalence of diseases.

One of the striking current trends is the gradual decrease on the prevalence of BN. Its place is being taken over by other disorders, and so the spectrum of EDs is widening. This points to the fundamental role of the sociocultural background and implies the need for social and health-policy action in prevention.

Variations in the presentation of EDs pose a challenge for psychiatry. The widening of the spectrum of EDs and body image disorders seems to be shifting mainly in the direction of body dissatisfaction. A further indication of the variability of conditions is the increasing frequency of association with impulse control disorders, a set of phenomena classified as the multi-impulsive subtype of EDs (Lacey & Evans, 1986).

The most striking observation that derives from our present results is *the change in body weight*. The spread of irregular forms of nutrition is one likely cause of the significant increase in average body weight among both women and men during the twenty-year period. For women, there is also a greater discrepancy between actual and desired body weight, and this may be expected to cause substantial psychological pressure. The increase in average body weight may also explain why the proportion of women in the BMI range 17.6–18.9 has decreased. In the thirty years after 1980, BMI in Central Europe among the general population increased at the rate of 0.4 kg/m^2^ per decade, and in Eastern Europe, at 0.2 kg/m^2^ (Finucane, Stevens, Cowan, Danaei, Lin & Paciorek, 2011). Our results are also consistent with the findings of a longitudinal study by Keel et al. (2006; 2007), which also found a rise in body weight in the period 1982–2002.

*The frequency of bingeing decreased* over the two decades, and among women, the change was significant. This may be related to the trend of decreasing BN incidence over recent decades (Smink et al., 2016; van Son, van Hoeken, Bartelds, van Furth & Hoek, 2006).

There has also been a considerable *decrease in the occurrence of irregular menstruation*, from 34.7 to 23.8% (*P* < 0.001). This decrease may be related to the more common use of oral contraceptives. That is why amenorrhoea no longer appears among the basic symptoms of AN defined in DSM-5 (American Psychiatric Association, 2013).

A remarkable finding from the ANIS data is the *increase in the risk of EDs among men* over the twenty-year period. The frequency of counterregulatory behaviours among men has also risen. The clinical and subclinical proportion of AN among male medical students also showed an increase, although this was not significant. The same trend is shown by the significant increase in the EDI drive for thinness subscale score among men. Our findings do not clearly confirm data pointing to the rise in EDs among men (Hudson, Hiripi, Pope, & Kessler, 2007; Strother, Lemberg, Stanford & Turberville, 2012), but they indicate an increasing risk. The size of our sample is insufficient to draw a definite conclusion in this respect.

Although the surveys found no AN of clinical severity among female medical students in either 1989 or 2011, they do show a *significant increase in the frequency of subclinical forms*. This is consistent with the findings of Lucas, Beard, O'Fallon and Kurland (1991), although their study concerned the incidence of EDs. They examined incidence data for AN and similar conditions going back fifty years. They ascribed the increase in incidence primarily to the proliferation of mild, subclinical forms. Sociocultural factors may have a greater influence on these than on clinical AN, the incidence of which did not change much and whose causes were considered by the authors to be mainly biological. They therefore distinguished type I (severe) and type II (mild, and mainly occurring through social pressure) AN (Lucas, 1992).

The significant changes in some EDI subscales may be due to *changes in gender roles*. Perfectionism scores increased among women, and ineffectiveness scores among men. These changes underline the complex nature of EDs, which are not merely a matter of eating and body weight but demand a broader study embedded in the social context.

The most similar study to ours was published by Weigel et al. (2016) from East Germany. The subjects were medical students and compared the situation before and after the political change. The intensive adaptation to Western beauty ideals was evident among females. This is corroborated by our study as well.

A key question in our research is how factors specific to geographical regions are relevant to mental disorders and EDs. A review of the psychosocial wellbeing of populations in Central and Eastern Europe (CEE) during the transition period, subsequent to the fall of the Soviet Bloc, found that subjective mediators such as locus of control, perceived control, self-efficacy beliefs, perceived familial support, and the subjective evaluation of social change explain part of the relationship between macrosocial changes and emotional wellbeing (Eiroá Orosa, 2013).

After the great sociocultural and political changes of 1989, one of the predominant themes in CEE countries was transition to the Western-style market economy and individualism. Among the consequences was the dwindling of social contacts (Rathner, 2001). In this respect, the study presented here may be regarded as part of a natural sociological experiment: the first phase of the study took place before the transition, and the second phase afterwards. Some authors suggest that EDs are sensitive barometers of cultural change (DiNicola, 1990; Nasser, 1997; Rathner et al., 1995).

### Limitations

Our study was made on a special, non-representative population of medical students. The extent to which they differ from the general student population, and whether the choice of a medical career involves special risks, remains open to question. In particular, we do not know the extent to which medical students constitute a population of high risk for EDs. Medical students have been found to be vulnerable to various mental health problems, such as depression and anxiety, due to the high workload of medical school (Ahmed, Banu, Al-Fageer & Al-Suwaidi, 2009).

Another possible limitation is that the 1989 sample was collected only from Debrecen, while the sample collected in 2011 was from Debrecen and Budapest. As Hungary is a small country, a relevant sociocultural difference cannot be expected between the two cities. Moreover, the data collection in Budapest was carried out using a mixed method design (paper-pencil and online questionnaires), which makes comparability less feasible.

Epidemiological studies of EDs face several methodological problems. These must be heeded when evaluating the results. The low prevalence of EDs of clinical severity in the general population presents a great difficulty, as do patients' denial of the disease and avoidance of medical assistance. Hoek (2006) estimated that 43% of patients with AN seek primary care and 34% specialised care; the figures for patients with BN are 11 and 6% respectively. It is uncertain whether the increasing number of patients presenting to healthcare settings indicates a real increase in the prevalence of EDs or an improved recognition of the conditions or the greater propensity of patients to seek help. It is also possible that survey respondents are more willing to speak openly about their problems, and this increases their visibility.

Several targets may be identified for future research. Changes in the characteristics of disorders linked to sociocultural factors should be tracked in view of the rapid historical changes. Medical students' attitudes and psychopathological risk factors should be compared with those of the general population and other students. This would give us greater insight into profession-specific factors. It would also be very important to recognise the new types of disorder that are appearing in the highly variable spectrum of EDs, even if these have not been assigned a place among official diseases in the nosological systems.

## Funding sources

The study was supported by the Hungarian Ministry of Health, Health Scientific Council: ETT (453/2009).

## Authors' contribution

FT: study concept and design, study supervision. PS: study concept and design, analysis and interpretation of data. SDS: analysis and interpretation of data. IS: analysis and interpretation of data. DS: statistical analysis. GR: study concept and design. All authors had full access to all data in the study and take responsibility for the integrity of the data and the accuracy of the data analysis.

## Conflict of interest

The authors declare no conflict of interest.
